# Berlin Letter

**Published:** 1884-11

**Authors:** 


### Foreign Correspondence.

BERLIN, October 7, 1884.

*Editors Buffalo Medical and Surgical Journal:*

Having followed, with considerable interest, the many investigations and consequent theories as to the cause of cholera, it seems to me that the conclusions of Dr. Koch are open to very serious objections. It is only reasonable to suppose that the bacilli, which he claims to be the essential cause of the disease in man, when injected into animals, should induce similar if not identical symptoms; but his efforts in this direction have thus far failed to produce the least indications of blood-poisoning. This has been urged by many as a proof that animals are insusceptible to the malady. In like manner the incredulous claim that not the bacillus, but, rather, some product evolved by it, is the true cause of the disease. This certainly seems probable enough to demand further investigation, and until some effect can be produced upon the higher animals by the injection of poisonous matter into their system, I do not think the real cause of the disease has been discovered.

The actual poison may exist at some particular step in the growth and development of the bacillus. Dr. Koch has not, as yet, traced its entire life-history, and, therefore, has not watched the many changes which it undoubtedly undergoes during the spore-bearing period. If we grant the same life-history to the bacillus previous to sporification which characterizes other such organisms, and, indeed, the forms which the same organism assumes are so different that they have often been assigned to distinct species, then it is not improbable that the cholera germ may exist in one of the more developed stages of the bacillus.

The recent discoveries of Drs. S. Maurin and Lange, who have been working at Marseilles, have convinced them that the actual agent in the propagation of cholera is a mucor. This mucor, they say, is the mature form of which the bacillus is an earlier and lower phase. Their report seems to indicate that it makes its appearance only on the fourth or fifth day, and, when visible, has the form of a mycelium whose tapering ends are surmounted by cup-shaped sporangia. The slightest agitation causes these sporangia to burst, thus discharging vast numbers of spores. Contact with some putrid organic matter germinates the spores, and they then develop into a mucor of another form, an anærobe, which they believe to be the immediate cause of the disease. The sporification of the anærobe gives birth to the bacilli of Dr. Koch. They claim to have proved, beyond a doubt, that the bacilli themselves are inocuous, but when deposited on a putrid medium and exposed to the air, they develop the first-mentioned mucor, and the cycle is renewed.

A rabbit, after being injected with these mucors, died in twenty-four hours with lesions entirely like those of cholera. The alleged difference between the mucor and the bacillus is the power of the former to resist disinfectants. Ten per cent. of nitric or hydrochloric acid has comparatively little effect upon it, while in a solution of carbolic acid of like proportions it vegetates freely. It withstands any temperature up to 150 degrees Centigrade, but above this breaks and disappears.

Strange as these statements may seem, there is really nothing in them save the power of resisting reagents, which is not in perfect harmony with what we know of the life-history of similar organisms. Very sincerely, L.

